# Shape control of moderately thick piezoelectric beams

**DOI:** 10.1007/s00707-023-03539-w

**Published:** 2023-03-31

**Authors:** Juergen Schoeftner

**Affiliations:** Institute of Technical Mechanics, Johannes Kepler University of Linz, Altenberger Str. 69, 4040 Linz, Austria

## Abstract

The present contribution focuses on shape control of thick beam-type structures. First the governing equations of a multi-layered beam are derived by taking advantage of the Timoshenko assumptions and the constitutive relations of piezoelectric materials. The deflection curves are explicitly given for a piezoelectric cantilever subjected to a polynomial distribution of the vertical load and the applied electric voltage. In order to find a solution for the optimal shape control voltage an objective function, which depends on the quadratic deflection curve over the beam length, is minimized. Finally several benchmark examples are given for thick beams and the outcome is compared to finite element results and previously derived shape control results from the scientific literature that hold for thin piezoelectric beams. The presented shape control method shows a better agreement with the numerical outcome than the analytical shape control results within the Bernoulli-Euler theory, but the desired voltage distribution only slightly differs from the outcome for thin beams. Furthermore it is found that for a given total thickness-to-length ratio piezoelectric bimorph structures may be more difficult to be perfectly controlled than three-layer beams with thin piezoelectric layers. This is due to higher order piezoelectric effects which are not considered by the present theory (e.g. the thickness deformation caused by the thickness piezoelectric coupling constant).

## Introduction

Mechanical constructions equipped with multi-functional materials that allow for manipulating the deflection and the motion in a desired manner are denoted as intelligent or smart structures. Piezoelectricity is one of several physical effects to control the deflection by proper actuation. Energy transfer takes place from the mechanical into the electrical domain by means of piezoelectric control devices. In case of passive vibration control or damping the energy is either dissipated in the electric circuit or stored (energy harvesting). An overview of this research topic, also denoted as adaptronics or structronics, is presented by Janocha [1]. Classical introductions to the field of piezoelectricity and vibration control are the books by Crawley [2], Miu [3], Tzou [4], Moheimani and Fleming [5] and Mura [6].

Piezoelectric transducers are well-established devices in vibration control, energy harvesting, sensing and structural health monitoring. In this contribution the main focus is laid on vibration control, in particular on shape control. This notion describes a special displacement tracking technique where one intends to completely annihilate (or strongly reduce) structural deflections. An excellent literature overview on shape control is presented by Irschik [7]. Shape control has been first introduced by Hafka and Adelman [8] who derived an analytical computation of the temperature field of a supporting structure to reduce distortions of space structures from their original shape. Vibrations of rotary wings were attenuated in Nitzsche and Breitbach [9], where smart devices were used to construct geometric modal filters that are able to control some critical modes. Austin et al. [10] designed adaptive wings, which included actuators to minimize the aerodynamic performance. Agrawal and Treanor [11] minimized a quadratic cost function of an unloaded shear-rigid cantilever, which contains the error between desired and achieved static deflection, to obtain the best locations for piezoceramics actuators. Shirazi et al. [12] designed a robust controller for tracking the tip deflection of a piezoelectric cantilever. A reduced order model was assumed for the cantilever via Galerkin’s method and the effects of the uncontrolled modes on the overall performance were studied. A planar hexagonal reflector was controlled by 30 PZT-actuators in Song et al. [13]. The proposed shape control method was also experimentally verified and considered model errors and uncertainties. The TLBO-method (teacher-learning based optimization) was implemented in the software code of COMSOL Multiphysics, which was used to model a piezoelectric bimorph, to nearly avoid structural deflections, see Sumit et al. [14]. In a further study [15] several optimization techniques were compared (simulated annealing (SA), genetic algorithm (GA), particle swarm optimization (PSO) and TLBO) for the same benchmark problem, finding that the SA method gave better values of the objective function and converged faster. Locations and actuation voltages of a cantilever plate are optimized by genetic algorithm by Wang et al. [16]. The deflections of laminated composite hybrid plate under thermo-electro-mechanical loads were investigated in Gohari et al. [17]. Assuming Kirchhoff kinematics, the plate was then controlled based on a double integral multivariable transformation method. Results were compared and verified against those available in the literature. Bendine and Wankhade [18] used a first order shear deformation theory and computed the required voltage via an adopted genetic algorithm to maintain a desired shape of the beam varying both loading and boundary conditions. If a piezoelectric layer or a multilayer beam with attached piezoelectric transducers is modeled within the framework of Bernoulli-Euler one achieves total elimination of the transverse deflection if the quasi-static bending moment of the smart control devices is equal, but sign-reversed to the quasi-static moment caused by the external forces, see Irschik et al. [19]. This result was experimentally verified by Nader [20]: relative deflections of a support-excited beam with attached piezoelectric patches are attenuated if the patches are properly placed and voltage-actuated. Irschik and Pichler et al. [21] showed that the distribution of the actuating stress has to be equal to the statically admissible stress to avoid vibrations of linear elastic structures. It is interesting to note that for statically indeterminate beams no deformation will occur if the electrodes of the piezoelectric layers are properly shaped in case of electrical actuation. Similarly, one may measure no voltage signal in case of sensing: this holds e.g. for a clamped-clamped beam with a constant distribution of the piezoelectric layer, see Hubbard and Burke [22]. These distributions are nil-potent shape functions, see Irschik et al. [23, 24] and [21], i.e. no matter how the voltage control signal is chosen, deformations will not occur. For thicker beams certain subsections are controlled by Krommer [25]. In [26] Krommer and Irschik consider both shear and extension actuation mechanisms and analytically showed that perfect annihilation of vibrations is possible. The role of the electrical boundary conditions at the vertical faces is studied by Krommer and Irschik [27], where a weak form of the charge equation of electrostatics is solved to find a solution for the electric potential distribution. In a previous work of them, they investigated if either the assumption of a vanishing electric displacement field or electric field in axial direction (i.e. $D_x = 0$ or $E_x = 0$ holds) gives a better correlation to finite element results, Krommer and Irschik [28]. For Reissner-Mindlin plate considering piezo- and pyroelectricity, the direct piezoelectric and the pyrelectric effects are incorporated in terms of effective stiffness parameters, see Krommer and Irschik [39]. It was found that the eigenfrequencies are higher for vanishing in-plane components of the electric displacement field than those for vanishing in-plane components of the electric field. If the electrodes cannot be considered as perfect in a sense that the equipotential area condition is fulfilled, one uses the notion resistive or moderately conductive electrodes, see Buchberger and Schoeftner [29] and Schoeftner et al. [30, 31]. If the electrode resistivity varies along the beam length in a certain manner the bending vibrations may be also attenuated, see [32]. Instead of properly tuning the electrode resistivity, it is much easier to attach patches at certain locations onto the elastic substrate which are then connected via resistances causing a desired voltage drop. Hence the theoretical framework and the experimental realization for this kind of vibration control technique is demonstrated in [33]. For monofrequent harmonic excitations shape control is also possible if the width of the layers is proportional to the quasi-static bending moment distribution and if the attached inductive electric circuit is driven in resonance. This principle is similar to a perfectly tuned vibration absorber connecting the mechanical and the electrical domain. Boley’s iterative method is extended by Schoeftner and Benjeddou [38] in when the compatibility equations and the charge equation of electrostatics are solved simultaneously for each layer. As representative example a simply-supported piezoelectric bimorph is investigated with sinusoidal voltage and distributed loads. It is shown that the error between analytical results and the solution from two-dimensional plane stress results decreases with each iteration.

This contribution presents results for shape control of laminated piezoelectric beams by considering shear rigidity. The necessary electric voltage actuation is calculated by minimizing the mean square error of the deflection. As suggested by Krommer and Irschik in [26] results are compared to two-dimensional finite element results under plane stress assumptions. From a theoretical point of view concerning piezoelectric modeling and control aspects, the method presented here is a particular case of the results in [26] neglecting the shear piezoelectric mode $\tilde{e}_{15}$ and the in-plane electric displacement and electric field, i.e. $D_x \approx E_x \approx 0$. The equivalent single layer theory for piezoelectric composites is assumed because the elastic moduli of the layered beam are of the same order of magnitude. First the differential equations of a piezoelectric beam are derived based on Timoshenko’s kinematic assumption and the constitutive relations for PZT-5A. The indirect and the direct piezoelectric effect are considered by approximately solving the charge equation of electrostatics to find relations between electric displacement, electric field and the displacement field. In a next step one computes the electric field from the constitutive relations. Consequently, the electric potentials of the piezoelectric layers are obtained by integration. Then the shear force and the bending moment are calculated. The latter has not only contributions from the mechanical degrees of freedom, but also from the electric field. Inserting into Newton’s law one finds the beam differential equations. In order to solve the shape control problem, a performance criterion is set up: the goal is to calculate the voltage actuation in order to minimize the square of the residual deflection. Finally several examples are presented in order to verify the proposed shape control method with finite element calculations, where the focus is laid on thick or moderately thick beams, i.e. the thickness-to-length ratios are $\lambda = 1/5$ and $\lambda = 1/10$. Comparing the results to two-dimensional finite element results in MATLAB one observes that the consideration of the shear influence becomes more and more important if the thickness of the elastic core, which is usually a non-piezoelectric material, is much larger than the thickness of the piezoelectric layers.

## Equations of motions of a piezoelectric Timoshenko beam

This section is concerned with mathematical modelling of moderately thick piezoelectric Timoshenko beams. The theoretical framework considers finite shear rigidity of the cross section on the one hand, but ignores the in-plane components of the electric field and the electric displacement on the other hand. For more details on piezoelectric beam modelling, the reader is referred to [27] and [28].

### Kinematics, constitutive relations and Newton’s law

The cross section displacement of a beam can be written as

1 $$\begin{aligned} {}&{} { u(x,z) = z \psi (x) } \nonumber \\{}&{} w(x,z) = w_0(x)\end{aligned}$$

where $w_0(x)$ is the transverse displacement and $\psi (x)$ is the rotation angle. Note that the axial deflection $u_0(x)$ and the normal force are not considered in the present study. This approach yields for the strains

2 $$\begin{aligned} {}{} & {} {} \varepsilon _{xx} = z \psi _{,x}(x) \nonumber \\ {}{} & {} {} \gamma _{xz} = \psi (x) + w_{0,x} (x) \end{aligned}$$

PZT-5A is the piezoelectric material in this study. The constitutive relations read

3 $$\begin{aligned} {}&{} \sigma _{xx} = \tilde{C}_{11} \varepsilon _{xx} - \tilde{e}_{31} E_z \nonumber \\ {}&{} \sigma _{xz} = \tilde{C}_{55} \gamma _{xz} - \tilde{e}_{15} E_x \end{aligned}$$

where $\tilde{C}_{11}$ and $\tilde{C}_{55}$ are the effective values of the stiffness matrix at constant electric field and $\tilde{e}_{31}$ and $\tilde{e}_{15}$ are effective values of the components from the piezoelectric coupling matrix. Note that $\tilde{C}_{ij} \ne C_{ij}$ and $\tilde{e}_{ij} \ne e_{ij}$ hold, i.e. the effective value may not to be confused with the 3*D*-matrix components, see [34]. The electric field vector is the negative gradient of the electric potential ${\textbf {E}} = [ E_x, \, E_z ]^T = -[ \varphi _{,x}, \, \varphi _{,z} ]^T$ and reads for the lower and the upper layer

4 $$\begin{aligned}{} & {} E_{z}^{\textrm{low}} = \frac{ V(x) }{ h_p } + \frac{ \tilde{e}_{31}}{ \tilde{\kappa }_{33} } \left( \frac{ h_p + h_s }{ 2 } - z \right) \psi _{,x} \end{aligned}$$

5 $$\begin{aligned}{} & {} E_{z}^{\textrm{upp}} = -\frac{ V(x) }{ h_p } - \frac{ \tilde{e}_{31}}{ \tilde{\kappa }_{33} } \left( \frac{ h_p + h_s }{ 2 } + z \right) \psi _{,x} \end{aligned}$$

Integrating Eqs. (4) and (5) with respect to *z* one finds for the electric potential

6 $$\begin{aligned}{} & {} \varphi ^{\textrm{low}} (x, \,z) = -\frac{ V(x) }{ h_p } \left( z - \frac{h_s}{2} \right) - \frac{ \tilde{e}_{31}}{ \tilde{\kappa }_{33} } \left[ \frac{ h_p + h_s }{ 2 }\left( z - \frac{h_s}{2} \right) - \left( \frac{z^2}{2}-\frac{h_s^2}{8} \right) \right] \psi _{,x} \end{aligned}$$

7 $$\begin{aligned}{} & {} \varphi ^{\textrm{upp}} (x, \,z) = \frac{ V(x) }{ h_p } \left( z + \frac{h_s}{2} \right) + \frac{ \tilde{e}_{31}}{ \tilde{\kappa }_{33} } \left[ \frac{ h_p + h_s }{ 2 } \left( z + \frac{h_s}{2} \right) + \left( \frac{z^2}{2}-\frac{h_s^2}{8} \right) \right] \psi _{,x} \end{aligned}$$

**Fig. 1:**
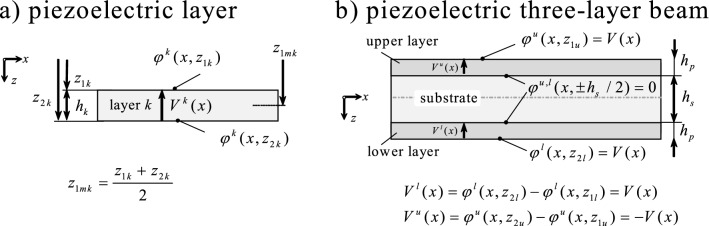
**a** piezoelectric layer *k* with electric potential $\varphi ^k(x,z)$, the thickness $h_k$ and the mean distance to the neutral fiber $z_{mk}$; **b** piezoelectric three-layer beam with thickness $h_p$, $h_s$ and symmetrical actuation $V^l (x) = -V^u (x) = V(x)$

Note that the electric potential at the substrate-layer interfaces is zero at $z = \pm h_s$: $\varphi ^{\textrm{low}} (x, \,h_s)$ $= \varphi ^{\textrm{upp}} (x, \,-h_s) = 0$. Here *V*(*x*) is the voltage drop of the prescribed potential distributions at $z = \pm h_s/2$ and $z = \pm (h_s/2 + h_p)$, see Fig. 1b.

The interested reader is referred to Appendix A for a more detailed derivation concerning the results for the electric field and the electric potential for upper and lower layers, see also Krommer [35] and Schoeftner [34]. Inserting Eqs. (4) and (5) into Eq. (3) one finds the bending moment and the shear force for a three-layer beam as

8 $$\begin{aligned}{} & {} M(x) = \int _{-c}^{c} \sigma _{xx} z { b } \, \textrm{d}z = K_M \psi _{,x} + C_M V(x) \end{aligned}$$

9 $$\begin{aligned}{} & {} Q(x) = \int _{-c}^{c} \sigma _{xz} { b } \, \textrm{d}z = K_Q \left( \psi + w_{0,x} \right) \end{aligned}$$

The bending stiffness is $K_M$ and the shear stiffness is $K_Q$. Note that the electric field $E_x$ is neglected for the shear force calculation in Eq. (9). However, the electric potential from Eqs. (6) and (7) could be differentiated with respect to *x* and inserted into Eq. (9). It is noted that taking into account the electric field $E_x \ne 0$ in this manner yields only slightly different results for shape control. From a modelling point of view and a detailed analysis on in-plane influence of $E_x$ or $D_x$, the reader is referred to Krommer and Irschik [27] and [28].

For the three-layer beam depicted in Fig. 1b, they read

10 $$\begin{aligned}{} & {} K_M = \tilde{C}_{11}^{s} \frac{ h_s^3 b }{ 12 } + \frac{ 2 }{ 3 } \tilde{C}_{11}^{p} \left[ \left( \frac{h_s}{2} + h_p \right) ^3 - \left( \frac{h_s}{2} \right) ^3 \right] b \end{aligned}$$

11 $$\begin{aligned}{} & {} K_Q = \left( \tilde{C}_{55}^{s} h_s b + 2 \tilde{C}_{55}^{p} h_p b \right) \kappa \end{aligned}$$

see Eqs. (42) and (43) when $z_{2\,s} = -z_{1\,s} = z_{1\,l} = -z_{2u} = h_s/2$ and $z_{2\,l} = - z_{1u} = h_s/2+h_p$ hold. The piezoelectric actuation constant $C_M$ reads

12 $$\begin{aligned} C_M = \tilde{e}_{31} \left( h_s+h_p \right) b \end{aligned}$$

The beam equlibrium requires that

13 $$\begin{aligned}{} & {} Q_{,x} + q(x) = I_{w} \ddot{w}_{0} \nonumber \\{} & {} \quad M_{,x} - Q(x) = I_{\psi } \ddot{\psi } \end{aligned}$$

where $I_{w}$ and $I_{\psi }$ are the mass and the moment of inertia per unit length. Inserting Eqs. (8) and (9) into (13) one obtains

14 $$\begin{aligned}{} & {} K_Q \left( \psi _{,x} + w_{0,xx} \right) + q(x) =I_{w} \ddot{w}_{0} \end{aligned}$$

15 $$\begin{aligned}{} & {} K_M \psi _{,xx} - K_Q \left( \psi + w_{0,x} \right) + C_M V_{,x} = I_{\psi } \ddot{\psi } \end{aligned}$$

## General solution of a piezoelectric Timoshenko beam

Static solutions of the piezoelectric Timoshenko beam equations for $\psi$ and $w_{0}$ are obtained by inserting $\psi _{,x}+w_{0,xx}$ from (14) into (15). One finds for the rotation angle by integrating the outcome three times with respect to *x*

16 $$\begin{aligned}{} & {} \psi (x) = -\frac{1}{l} \left( A_1 + \frac{6 K_M}{l^2 K_Q} A_3 + 2 A_2 \frac{x}{l} + 3 A_3 \frac{x^2}{l^2} \right) \nonumber \\{} & {} \quad \quad \quad \quad \; - \frac{ C_M }{K_M} \int V(x) \, \textrm{d}x - \frac{ 1}{K_M} \int q(x) \, \textrm{d}x^3 \end{aligned}$$

and for the deflection

17 $$\begin{aligned}{} & {} w(x) = A_0 + A_1 \frac{x}{l} + A_2 \frac{x^2}{l^2} + A_3 \frac{x^3}{l^3} + \frac{ 1}{ K_M } \int q(x) \, \textrm{d}x^4 \nonumber \\{} & {} \quad \quad \quad \quad \; - \frac{ 1 }{ K_Q } \int q(x) \, \textrm{d}x^2 + \frac{ C_M }{ K_M } \int V(x) \, \textrm{d}x^2 \end{aligned}$$

In the following we assume mechanical and electrical loads in the form of polynomials

18 $$\begin{aligned}{} & {} q(x) = q_0 + q_1 \frac{x}{Ll} + q_2 \frac{x^2}{l^2} + q_3 \frac{x^3}{l^3} + \ldots \nonumber \\{} & {} \quad V(x) = V_0 + V_1 \frac{x}{l} + V_2 \frac{x^2}{l^2} + V_3 \frac{x^3}{l^3} + \ldots \end{aligned}$$

Inserting (18) into (17) and (16) and determining the four coefficients $A_0,$ $A_1,$ $A_2,$ $A_3$ from the boundary conditions, one may easily find the solution of the piezoelectric beam. In the next subsection this is demonstrated for a piezoelectric cantilever.

### Solution of a piezoelectric cantilever

The boundary conditions for a cantilever with tip force $F_0$ read $w(0) = \psi (0) = M(l) = 0$ and $Q(l) = F_0$. The solution above can be split up into a load-dependent $w_q (x)$ and a voltage dependent part $w_V (x)$

19 $$\begin{aligned} w(x) = w_q (x) + w_V (x) \end{aligned}$$

Additionally, it is advantageous to split the mechanical part $w_q (x)$ into a fundamental term (=Bernoulli-Euler (BE) solution) and into a higher order (HO) solution $w_{HO}(x)$, which considers the influence of shear. In case of a tip force $F_0$ and a quadratic load distribution (i.e. $q_i = 0$ for $i \ge 3$) one finds

20 $$\begin{aligned}{} & {} w_q(x) = w_{BE} (x) + w_{HO} (x)\end{aligned}$$

21 $$\begin{aligned}{} & {} {} \text {with} \qquad w_{BE}(x) = \frac{ \left( 3-\xi \right) \xi ^2 }{6 K_M} F_0 l^3 + \frac{ \left( 6- 4\xi + \xi ^2 \right) \xi ^2 }{24 K_M} q_0 l^4 \nonumber \\{} & {} {} \qquad \qquad \; + \frac{ \left( 20 - 10\xi + \xi ^3 \right) \xi ^2 }{120 K_M} q_1 l^4 + \frac{ \left( 45 - 20\xi + \xi ^4 \right) \xi ^2 }{360 K_M} q_2 l^4 \end{aligned}$$

22 $$\begin{aligned}{} & {} w_{HO}(x) = \frac{ \xi }{K_Q} F_0 l + \frac{ \left( 2- \xi \right) \xi }{2 K_Q} q_0 l^2 + \frac{ \left( 3 - \xi ^2 \right) \xi }{6 K_Q} q_1 l^2 + \frac{ \left( 4 - \xi ^3 \right) \xi }{12 K_Q} q_2 l^2 \end{aligned}$$

Note that the non-dimensional variable $\xi = x/L$ is introduced. In case of voltage actuation one finds

23 $$\begin{aligned} w_V (x) = \left[ \frac{ \xi ^2 }{2 } V_0 l^2 + \frac{ \xi ^3 }{6 } V_1 l^2 + \frac{ \xi ^4 }{12 } V_2 l^2 + \frac{ \xi ^5 }{20 } V_3 l^2 + \frac{ \xi ^6 }{30 } V_4 l^2 \right] \frac{ C_M }{ K_M } \end{aligned}$$

It is noted that the solutions (19)–(23) include also the solutions of a clamped-hinged beam: for such a statically indeterminate structure the tip force $$F_0$$ needs to be adjusted such that $$w(l) = 0 $$ holds in Eq. (19). Consequently the shear force does not vanish $$Q(l) \ne 0$$.

## Shape control criterion: minimizing the quadratic error of the vertical deflection

The shape control problem is employed to minimize the quadratic error of the vertical deflection. The objective function is defined by

24 $$\begin{aligned} J = \int _{ 0 }^{ l } w^2 (x, \, q(x), \, V(x) ) \, \textrm{d}x \end{aligned}$$

The goal is to minimize the error function *J* for a given load distribution *q*(*x*) by properly adjusting the voltage distribution *V*(*x*). In a first step the optimization problem (24) is simplified: a polynomial is assumed for the load (e.g. $q(x) = q_0 +q_1 x/l$), similarly also for the voltage distribution (e.g. $V(x) = V_0 + V_1 x/l + V_2 x^2/l^2 + V_3 x^3/l^3 + V_4 x^4/l^4$). The set of control variables is $C = \{ V_0, \, V_1, \, V_2, \, V_3, \, V_4 \}$. Hence the objective function of the modified shape control problem is rewritten

25 $$\begin{aligned} J = \int _{ 0 }^{ l } w^2 (x, \, q_0, \, q_1, \, C ) \, \textrm{d}x \end{aligned}$$

and minimized

26 $$\begin{aligned} \min _C \, J = \min _C \int _{ 0 }^{ l } w^2 (x, \, q_0, \, q_1, \, C ) \, \textrm{d}x \end{aligned}$$

This leads to an optimization problem with five variables to be solved, namely $V_0, \, V_1, \, V_2, \, V_3, \, V_4$. Inserting Eqs. (20)–(23) into Eq. (26) one finds

27 $$\begin{aligned} \min _C \int _{ 0 }^{ l } \left[ w_{BE}^2 + w_{HO}^2 + 2 w_{BE} w_{HO} + 2 \left( w_{BE} + w_{HO} \right) w_{V} + w_{V}^2 \right] \, \textrm{d}x \end{aligned}$$

Partial derivation of (27) with respect to the yet unknown voltage coefficient $V_i$ yields five equations, from which the unknowns may be computed

28 $$\begin{aligned}{} & {} \frac{ \partial J}{ \partial V_0} = \int _{ 0 }^{ l } \left( w_{BE} + w_{HO} + w_{V} \right) \frac{ \partial w_{V} }{ \partial V_0 } \textrm{d}x = 0 \nonumber \\{} & {} \quad \frac{ \partial J}{ \partial V_1} = \int _{ 0 }^{ l } \left( w_{BE} + w_{HO} + w_{V} \right) \frac{ \partial w_{V} }{ \partial V_1 } \textrm{d}x = 0 \nonumber \\{} & {} \quad \frac{ \partial J}{ \partial V_2} = \int _{ 0 }^{ l } \left( w_{BE} + w_{HO} + w_{V} \right) \frac{ \partial w_{V} }{ \partial V_2 } \textrm{d}x = 0 \nonumber \\{} & {} \quad \frac{ \partial J}{ \partial V_3} = \int _{ 0 }^{ l } \left( w_{BE} + w_{HO} + w_{V} \right) \frac{ \partial w_{V} }{ \partial V_3 } \textrm{d}x = 0 \nonumber \\{} & {} \quad \frac{ \partial J}{ \partial V_4} = \int _{ 0 }^{ l } \left( w_{BE} + w_{HO} + w_{V} \right) \frac{ \partial w_{V} }{ \partial V_4 } \textrm{d}x = 0 \end{aligned}$$

Inserting Eqs. (21), (22) and (23) into (28) the coefficients $V_i$ are obtained.

Neglecting the higher order term (i.e. $w_{HO}(x) = 0 \rightarrow w(x) = w_{BE}(x) + w_V (x)$)

29 $$\begin{aligned} \frac{ \partial J}{ \partial V_i} = \int _{ 0 }^{ l } \left( w_{BE} + w_{V} \right) \frac{ \partial w_{V} }{ \partial V_i } \textrm{d}x = 0 \qquad i= { 0,\,} 1,\, 2,\, 3,\, 4 \end{aligned}$$

the solution for shape control of a Bernoulli-Euler beam is obtained, but without so-called nil-potent shape functions caused by static redundancy, see to Irschik et al. [19] and [21].

## Verification and comparison to finite element results—benchmark examples

In this section the presented shape control procedure for a piezoelectric Timoshenko beam is validated. Two simple examples are under consideration: first a piezoelectric cantilever (i.e. substrate and attached piezoelectric layers) which is subjected to a uniformly distributed load is considered. Then a piezoelectric clamped-hinged beam with linearly decreasing load is investigated which is an example for a statically indeterminate structure. Furthermore, a moderately thick (thickness-to-length ratio $\lambda = 1/10$) and a thick beam ($\lambda = 1/5$) are considered. The beam length is $l = 80 \, \textrm{mm}$ and the total beam thickness is $16 \, \textrm{mm}$ for the thick beam. If the substrate thickness ratio is $\lambda _s = 0.8$ (also called core ratio), the thickness of the substrate and of each piezoelectric layer are $12.8 \, \textrm{mm}$ and $1.6 \, \textrm{mm}$, respectively. For the thinner beam these values read $6.4 \, \textrm{mm}$ and $0.8 \, \textrm{mm}$. As already stated in the introduction, the shape control problem (24) has been extensively treated for thin beams within the framework of Bernoulli-Euler when total elimination of force-induced deflections for any given load distribution is possible, see e.g. [19] and [21]. For Timoshenko beams shape control has not been studied as systematically as for thin beams, see [25] and [26], hence this contribution also tries to close this gap. The finite element code is written in MATLAB. A Q8-element (eight-node quadrilateral element with quadratic ansatzfunctions for both displacement components and for the voltage) is used for the finite element calculations. The modified structure of this FE code is based on the book by Ferreira [36], who presents a huge variety of standard finite elements (beams, plates and two-dimensional elements) for elastic structures. For this research study the original code is adapted and piezoelectric properties are properly taken into account, see Piefort [37]. 8064 rectangular elements are used in case of a thick piezoelectric beam ($\lambda = 1/5$, 96 elements in the axial and 84 elements in the thickness direction). Hence the mean size-aspect ratio is 4.375, further mesh refinements and a lower aspect ratio do not significantly improve the result. It is noted that the element aspect ratio *AR* is not constant: 68 elements are used for the substrate in thickness direction ($h_s = 12.8 \, \textrm{mm}$ $\rightarrow \, AR_{subst} \approx 4.43$) and 8 elements are used for each piezoelectric layer ($h_p = 1.6 \, \textrm{mm}$ $\rightarrow \, AR_{piezo} \approx 4.17$). In case of electrical actuation and shape control, the electric voltage is evaluated from Eqs. (28) or (29) and prescribed at the voltage nodes at $z = \pm ( h_s/2 + h_p )$. The electrical nodes at $z = \pm h_s/2$ are grounded. For the thin beam with $\lambda =1 /10$ the number of elements in thickness direction is halved, hence the average aspect ratio remains the same.

The geometrical and material parameters for the substrate and the piezoelectric layers are summarized in Table 1.

**Table 1:** Parameters for the numerical examples

Variable (unit)	Value	Description
$l \,\quad (\textrm{m})$	0.08	Length
$\lambda \,\quad (\mathrm {-})$	(either 1/5 or 1/10)	Thickness-to-length ratio
$\lambda _s = h_s/(h_s+2h_p) \,\quad (\mathrm {-})$	(varies between 0 and 0.99)	Substrate thickness ratio (see Fig. 1b)
$\tilde{C}^{\textrm{p}}_{11} \,\quad (\textrm{Nm}^{-2})$	$6.098 \cdot 10^{10}$	Short-circuit elastic stiffness constant
$\tilde{C}^{\textrm{p}}_{55} \,\quad (\textrm{Nm}^{-2})$	$2.105 \cdot 10^{10}$	Short-circuit elastic stiffness constant
$\tilde{\kappa }^{\textrm{p}}_{33} \,\quad (\textrm{AsV}^{-1} \textrm{m}^{-1})$	$1.327 \cdot 10^{-9}$	Strain-free permittivity
$$\tilde{e}^{\textrm{p}}_{31} \,\quad (\textrm{Asm}^{-2})$$	$$-10.427 $$	Transverse piezoelectric coupling constant
$\tilde{C}^{\textrm{s}}_{11} = E \,\quad (\textrm{Nm}^{-2})$	$21 \cdot 10^{10}$	Young’s modulus (steel)
$\tilde{C}^{\textrm{s}}_{55} = E/[2(1+\nu )] \,\quad (\textrm{Nm}^{-2})$	$8.077 \cdot 10^{10}$	Shear modulus (steel with $\nu = 0.3$)
$q_0 = q_1 \,\quad (\textrm{Nm}^{-1})$	1	Value for load distribution

**Fig. 2:**
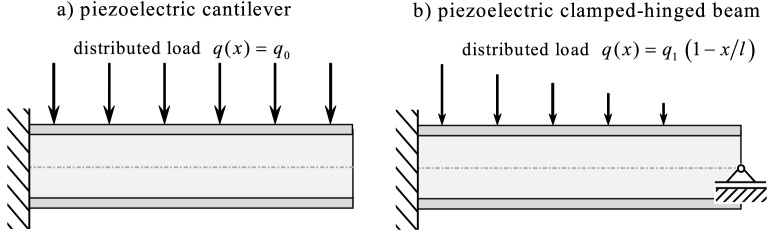
Benchmark examples for shape control of moderately thick beams: **a** cantilever with uniformly distributed load; **b** clamped-hinged piezoelectric beam with linearly decreasing load

### Example I: shape control of a cantilever with distributed load $q(x) = q_0$

**Fig. 3:**
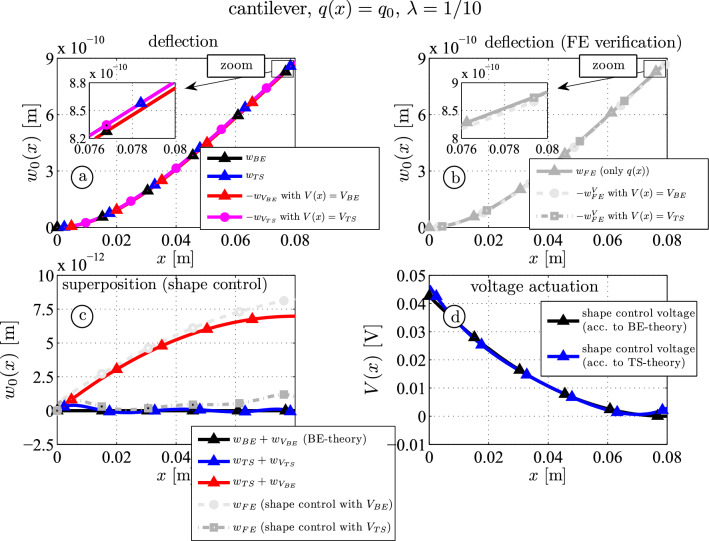
Moderately thick piezoelectric three-layer beam ($\lambda = 1/10$): analytical and numerical (FE) deflection curves due to mechanical and piezoelectric actuation (**a** and **b**); shape control results (**c**); shape control voltages according to the Bernoulli-Euler (BE) and the Timoshenko (TS) theory, see Eqs. (30) and (31) (**d**)

Fig. 2a shows the first benchmark example. First, results for a thin beam ($\lambda = 1/10$) are investigated, then for a thicker one ($\lambda = 1/5$). Figure 3a shows analytical results caused by mechanical and piezoelectric actuation of the vertical deflection:

- $w_{BE}$ (black curves): Bernoulli-Euler beam deflection due to $q(x) = q_0 = 1\, \mathrm {N/m}$ and $V(x) = 0 \,\textrm{V}$ according to Eq. (21) (mechanical actuation).
- $w_{TS}$ (blue curves): Timoshenko beam deflection due to $q(x) = q_0 = 1\, \mathrm {N/m}$ and $V(x) = 0 \,\textrm{V}$ according to Eq. (20) (mechanical actuation).
- $w_{V_{BE}}$ (red curves): Bernoulli-Euler beam deflection due to $q(x) = 0\, \mathrm {N/m}$ and $V(x) \ne 0 \,\textrm{V}$ according to Eq. (23) (piezoelectric actuation). The voltage actuation is calculated according to Eq. (29) or (30).
- $w_{V_{TS}}$ (red curves): deflection due to $q(x) = 0\, \mathrm {N/m}$ and $V(x) \ne 0 \,\textrm{V}$ according to Eq. (23) of a Timoshenko beam (piezoelectric actuation). The voltage actuation is calculated according to Eq. (28) or (31).

Figure 3b shows the finite element results for comparison:

- $w_{FE}$ (grey-triangle curves): deflection due to $q(x) = q_0 = 1\, \mathrm {N/m}$ and $V(x) = 0 \,\textrm{V}$ (mechanical actuation).
- $w_{FE}^V$ (light grey-circle curves): deflection due to $q(x) = 0\, \mathrm {N/m}$ and $V(x) \ne 0 \,\textrm{V}$ according to the voltage from Eq. (29) or (30) (piezoelectric actuation).
- $w_{FE}^V$ (grey-square curves): deflection due to $q(x) = 0\, \mathrm {N/m}$ and $V(x) \ne 0 \,\textrm{V}$ according to the voltage from Eq. (28) or (31) (piezoelectric actuation).

As already known from literature the mechanical and piezoelectric deflections using the Bernoulli-Euler shape control method from Irschik [19] (black and red curves) are equal, but opposite in sign, see Fig. 3a. Note that the results for the piezoelectric actuation ($w_{V_{BE}}$, $w_{V_{TS}}$, $w^{V}_{FE}$) are sign-reversed (see the legend in Fig. 3a and b). The necessary voltage actuation is proportional to the quasi-static bending moment distribution and reads according to Eq. (29)

30 $$\begin{aligned}&{} V_{BE}(x) = V_0 + V_1 x/l + V_2 x^2/l^2 \nonumber \\ {}&{} \text {with} \qquad V_0 = -\frac{q_0 l^2}{2 C_M}, \quad V_1 = \frac{q_0 l^2}{ C_M}, \quad V_2 = -\frac{q_0 l^2}{2 C_M}, \quad \end{aligned}$$

For the Timoshenko beam one obtains from Eq. (28)

31 $$\begin{aligned}{} & {} V_{TS}(x) = V_{BE}(x) + V_{HO}(x) \qquad \textrm{with} \qquad \end{aligned}$$

32 $$\begin{aligned}{} & {} V_{HO}(x) = \frac{ q_0 l^2}{C_M \alpha } \left( -\frac{ 43 }{ 2 } + 270 \frac{x}{l} - 990 \frac{x^2}{l^2} + \frac{ 9900 }{ 7 } \frac{x^3}{l^3} - \frac{ 19305 }{ 28 } \frac{x^4}{l^4} \right) \end{aligned}$$

The non-dimensional parameter $\alpha$ depends on the shear-bending stiffness ratio and the length

33 $$\begin{aligned} \alpha = \frac{ K_Q l^2 }{ K_M } \end{aligned}$$

The deflection curves (Fig. 3a) following the Bernoulli-Euler theory caused by the distributed load and the voltage actuation are denoted by $w_{{BE}}$ (black) and $w_{V_{BE}}$ (red), respectively, those following the Timoshenko theory are $w_{{TS}}$ (blue) and $w_{V_{TS}}$ (light blue). As already mentioned the superposition of the Bernoulli results $w_{{BE}} + w_{V_{BE}}$ vanishes (black curve in Fig. 3c). For the Timoshenko beam this is only approximately fulfilled, a very small residual error remains, see $w_{{TS}} + w_{V_{TS}}$ (blue). Superposing the Timoshenko loaded beam with the voltage actuation obtained from the BE-theory (30), $w_{{TS}} + w_{V_{BE}}$ (red), one observes a slightly larger error. These results can be verified by the two-dimensional finite element results which agree quite well with the analytical results based on Timoshenko’s theory: adding the deflection caused by the BE-shape control actuation (30) to the force-loaded beam (light grey circle) is close to corresponding analytical result $w_{{TS}} + w_{V_{BE}}$ (red). Using the TS-shape control voltage distribution (31) yields a negligible deflection (dark grey square, cf. analytical result (blue)). The shape control voltages from (30) and (31), which are almost equal, are shown in Fig. 3d for comparison.

**Fig. 4:**
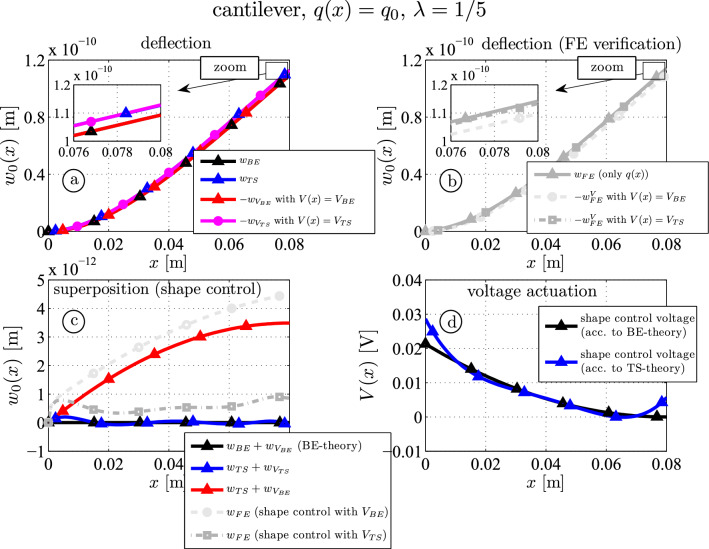
Thick piezoelectric three-layer beam ($\lambda = 1/5$): analytical and numerical (FE) deflection curves due to mechanical and piezoelectric actuation (**a** and **b**); shape control results (**c**); shape control voltages according to the Bernoulli-Euler (BE) and the Timoshenko (TS) theory, see Eqs. (30) and (31) (**d**)

For the thicker beam ($\lambda = 1/5$) the results are shown in Fig. 4. Qualitatively the outcome is very close to the results for the thinner beam, but the influence of the beam thickness becomes obvious. The residual deflection for the thick beam is about $3.2\%$ at $x=l$ (red, Fig. 4c), while the mean residual deflection for the thin beam ($\lambda = 1/10$, Fig. 3c) is about $0.8\%$ of the non-controlled deflection if the BE-shape control voltage is applied. The FE results also show a similar curve if the BE-shape control voltage is applied (compare red and light grey curves). Although some other higher order influences come into play (e.g. the transverse normal stress $\sigma _{zz}$ or strain $\varepsilon _{zz}$ and the transverse piezoelectric coupling constant $e_{15}$), which are negligible for thinner beams, the agreement between analytical and numerical shape control results is very good if TS-shape control voltage is applied: the residual deflection varies around zero for the Timoshenko beam (blue) and only $0.8\%$ ($8.6 \cdot 10^{-13} \textrm{m}$ at $x=l$) for the FE-model (compare blue and dark grey curves). Furthermore, the influence of the thickness-ratio on the voltage actuation, see Eq. (32), according to BE and TS-theory is more visible (cf. Figs. 3d and 4d). Although even for most practical problems the remaining deflection is small if the BE-shape control voltage is used, the influence of shear is present and can be considered by the Timoshenko actuation voltage (31), which may be important for high precision control.

#### Variation of the core thickness

**Fig. 5:**
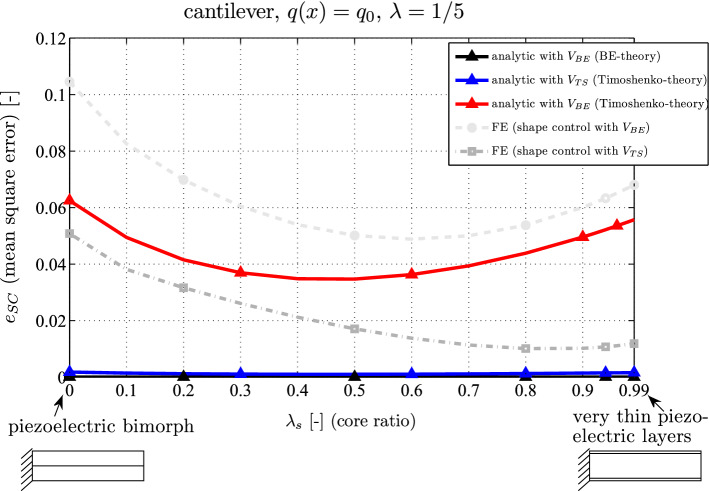
Relative mean square error $e_{SC}$ in case of shape control for a piezoelectric cantilever (analytical and FE results as a function of the core thickness ratio $\lambda _s$)

In the previous section 5.1 the relative thickness of the substrate is $\lambda _s = 0.8$. Figure 5 shows the residual shape control deflection as a function of the substrate thickness. Hereby, the relative mean square error $e_{SC}$ is calculated in the following manner

34 $$\begin{aligned} e_{SC} = \sqrt{ \frac{ \int _0^l \left( w_{a} + w^V_{a} \right) ^2 \, \textrm{d}x}{ \int _0^l w_{a}^2 \, \textrm{d}x } } \qquad a = BE \,\, \textrm{or} \,\, TS \end{aligned}$$

where the tracer *a* means Bernoulli-Euler (BE) or Timoshenko (TS). Assuming a thick beam again ($\lambda = 1/5$) a piezoelectric bimorph is obtained if the substrate is neglected ($\lambda _s = 0 \rightarrow h_s = 0\, \textrm{mm}$, $h_p = 8\, \textrm{mm}$). If the substrate thickness ratio is $\lambda _s = 0.99$, the piezoelectric layers are very thin ($\lambda _s = 0.99 \rightarrow h_s = 15.84\, \textrm{mm}$, $h_p = 0.08\, \textrm{mm}$).

One observes again that shape control following the BE-theory yields a perfect annihilation of the deflection (black), but this only holds for very thin beams. Assuming the TS-theory and using the voltage actuation (31) the remaining deflection is also close to zero for thick beams (blue). Applying the BE-voltage (30) on the TS-loaded beam yields a residuum between $3.5\%$ and $6.2\%$ of the uncontrolled deflection (red), but the numerical result from FE-model shows a similar distribution and is close to it for $\lambda _s > 0.6$ (light grey circle, between $4.9\%$ and $10.5\%$). Using the TS-shape control voltage (dark grey square) the numerical outcome converges to the analytical outcome (blue) if the substrate thickness is larger and the layer thickness thinner.

### Example II: shape control of a clamped-hinged beam with distributed load $$q(x) = q_0 ( 1-x/l ) $$

**Fig. 6:**
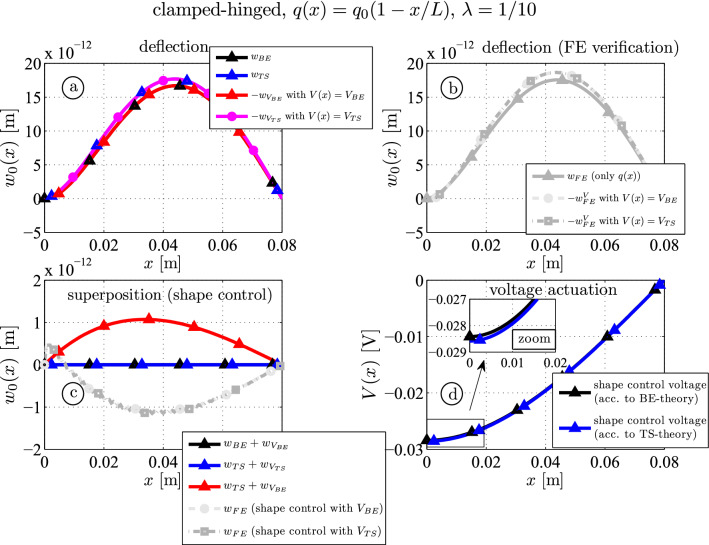
Moderately thick piezoelectric three-layer beam ($\lambda = 1/10$): analytical and numerical (FE) deflection curves due to mechanical and piezoelectric actuation (**a** and **b**); shape control results (**c**); shape control voltages according to the Bernoulli-Euler (BE) and the Timoshenko (TS) theory, see Eqs. (35) and (36) (**d**)

**Fig. 7:**
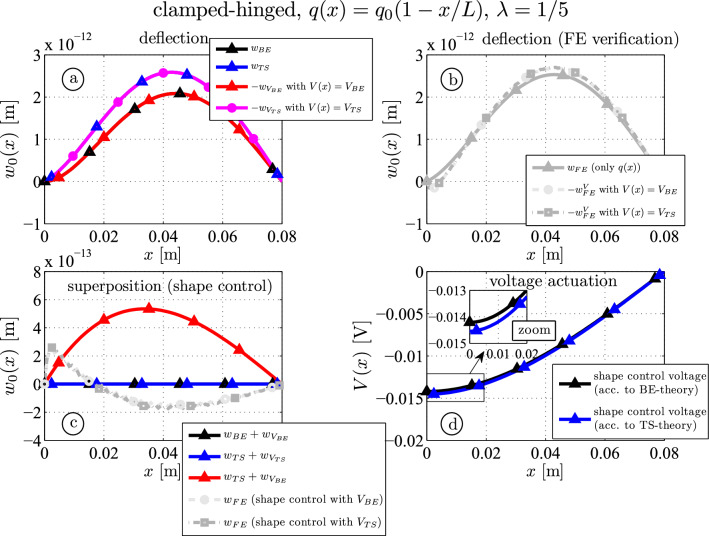
Thick piezoelectric three-layer beam ($\lambda = 1/5$): analytical and numerical deflection (FE) curves due to mechanical and piezoelectric actuation (**a** and **b**); shape control results (**c**); shape control voltages according to the Bernoulli-Euler (BE) and the Timoshenko (TS) theory, see Eqs. (35) and (36) (**d**)

The second example shows a clamped-hinged beam subjected to the linearly decreasing load $$q(x) = q_0 ( 1-x/l ) $$. Again a thin beam (Fig. 6) and a thick beam (Fig. 7) are considered. The results from Irschik [19] show that for statically indeterminate beams so-called nil-potent shape functions exist which do not cause any deflections. The shape control voltage reads

35 $$\begin{aligned} V_{BE}(x) = \frac{q_0 l^2}{6 C_M} \left( 2- 3\frac{ x^2 }{ l^2 } + \frac{ x^3 }{ l^3 } \right) + V_{np} \left( 1 - \frac{ x }{ l } \right) \end{aligned}$$

where the second part does not affect the solution, i.e. the constant $V_{np}$ is arbitrary. It is noted that the solution according to the minimization procedure presented in Sect. 4 yields

36 $$\begin{aligned} V_{TS}(x) = \frac{q_0 l^2}{6 C_M} \left( 2- 3\frac{ x^2 }{ l^2 } + \frac{ x^3 }{ l^3 } \right) + \frac{q_0 l^2}{ C_M \alpha } \left( 1- \frac{ x }{ l } \right) \end{aligned}$$

In case of a thin beam, when $\alpha \rightarrow \infty$ holds, Eqs. (35) and (36) match if the linear nil-potent contribution is disregarded $V_{np} = 0$.

The results for the deflection of a thin piezoelectric beam are shown in Fig. 6. Analytical and numerical results caused by mechanical or electrical actuation are shown in Fig. 6a and b. Superposition of both types of actuation yields the residual shape control deflection, see Fig. 6c. This time one observes that perfect annihilation can be also achieved by the Timoshenko model (blue). Calculating the shear force by inserting (16), (17) and (36) into (9) one finds a vanishing reaction force at the clamped end $Q(0) = 0$ caused by the lower order term on the right side of (36). This result is also obtained by the 2D finite element model where $\int _{-c}^c \sigma _{xz} \, \textrm{d}z = 0$ holds, but locally $\sigma _{xz} \ne 0$ holds which cause deformations close to the clamped end.

For statically redundant beams the reaction force can be manipulated such that the superposition of mechanically and voltage loaded beams becomes zero. This is quite impressive because differences of the necessary shape control voltages by the TS- and the BE-model are rather small, see the zoomed part in Fig. 6d. Using the BE-shape control voltage (35) for the Timoshenko model yields a maximum deflection of $1.1 \cdot 10^{-12}\, \textrm{m}$ (red), which is $6.2\%$ of the maximum deflection without control. The FE outcome yields a similar results, but sign-reversed, no matter if $V_{BE} (x)$ or $V_{TS} (x)$ is applied over the surface of the piezoelectric layers.

For the thick clamped-hinged piezoelectric beam the deflections of the Timoshenko beam are cancelled if (36) is applied over the piezoelectric surface, see Fig. 7c. Using the BE-shape control voltage (35) the residual deflection is $5.3 \cdot 10^{-13} \textrm{m}$ at $x=0.034\, \textrm{m}$, which is approximately $20\%$ of the maximum deflection without control. The FE results show a smaller residual deflection, but the difference between the chosen shape control voltage is negligible. It is noted that the results of the numerical simulation show that the thickness deformation (mainly caused by the thickness piezoelectric mode $e_{33}$) has the same order of magnitude as the residual error. Additionally, close to the clamped end at $x=0$ (Fig. 7b and c) one observes influences in case of piezoelectric actuation.

#### Variation of the core thickness

**Fig. 8:**
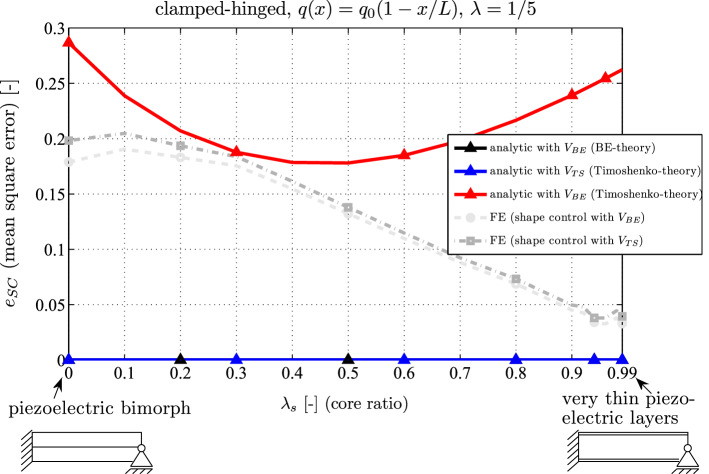
Residual shape control deflection (relative mean square error) of a clamped-hinged beam for analytical and FE results as a function of the core thickness ratio $\lambda _s$

In Fig. 8 the residual shape control deflection is plotted as a function of the substrate thickness. One observes that shape control can be achieved according to the BE or the TS theory if the corresponding shape control voltages are applied. Using the BE-voltage in the Timoshenko model yields a mean deflection varying between 18 and 29% compared to the uncontrolled clamped-hinged piezoelectric beam. The numerical results show no significant differences between both shape control voltages: shape control for a piezoelectric bimorph ($\lambda _s = 0$) yields a residual deflection of $20\%$; if very thin piezoelectric layers are used shape control is rather efficient and the deflection is only $3 \%$ of the uncontrolled deflection. This graph reveals that other piezoelectric coupling constants play a dominant role beside the transverse one ($e_{31}$) (e.g. the shear $e_{15}$ and the thickness mode $e_{33}$) unless the piezoelectric layers are thin. In case of thin piezoelectric layers (as it is the case for $\lambda _s = 0.99$ even if the total thickness is large (e.g. $\lambda =1/5$)), the dominant piezoelectric effect is the transverse mode, thickness and shear effects can be disregarded.

Hence, it is inevitable to consider these coupling effects if one intends to improve analytical piezoelectric models for thick laminate composites in the future. Nevertheless, from a practical point of view using the BE-theory for shape control seems to be a good choice for at least two reasons:

- the difference between the shape control voltage of BE and TS is small. For practical reasons it is advantageous to use so-called resistive electrodes or perfectly placed piezoelectric layers and an approximated discretized voltage actuation in order to replace the ideal continuous distribution, see (36).
- A more accurate analytical piezoelectric beam theory used for shape control must consider also the thickness deformation and the corresponding piezoelectric coupling constant $e_{33}$.
- The exact realization of the mechanical boundary conditions (e.g. a clamped end can be realized by $u(0,z) = w(0,z) = 0$ or $w(0,z) = u(0,0) = \int _{-h_s}^{h_s} u(0,z) = 0$ in the FE model) has a lower order impact on the deflection, which is in the range of the residual deflection. Hence a higher order analytical piezoelectric model may also consider (beside higher order piezoelectric effects due to $e_{15}$ and $e_{33}$) this influence in order to be more accurate.

To the best knowledge of the author, no higher order analytical piezoelectric beam theory exists which takes into account these last two points.

## Conclusion

In this contribution shape control is investigated for thick beams. Based on the kinematic assumptions of Timoshenko and on the constitutive relations for piezoelectric materials, the differential equation of a multilayer beam are derived. For a cantilever the general solutions for the deflection curve and the cross-sectional rotation are given in case of mechanical actuation (i.e. distributed load and tip force) and electrical actuation. By minimizing an objective criterion which depends on the quadratic deflection over the beam length for a certain polynomial load distribution, the optimized piezoelectric voltage distribution is obtained. Finally the outcome is compared to two-dimensional finite element calculations which is considered as target solution. Two examples are considered: a moderately thick ($\lambda =1/10$) and a thick ($\lambda = 1/5$) three-layer beam (two piezoelectric layers and an elastic substrate) with clamped-free and clamped-hinged boundary conditions are investigated. Based on the given distribution of the mechanical load, the optimized voltage is calculated in order to perform shape control. A special result is obtained by neglecting the influence of shear, where the output is compared to previously obtained results from the scientific literature. The deflection curves are shown for mechanical load and electrical actuation and their superpositions. It is found that the Timoshenko outcome for shape control is in better agreement to the target solutions as the Bernoulli-Euler results. A parameter study where the relative thickness of the substrate is varied yields that the agreement between analytical and finite element results is much better if the piezoelectric layers are relatively thin as compared to a piezoelectric bimorph (i.e. if the total thickness-to-length ratio remains constant) because higher order effects like deformations due to the shear and the thickness piezoelectric mode are not considered by the present theory. Nevertheless, from a practical point of view it is found that the shape control theory according to the Bernoulli-Euler theory seems to yield a sufficient reduction of the deflection.

## A Charge equation of electrostatics for layer *k* and forces and moments on beam level

Consider both the direct and indirect piezoelectric effect, the charge equation of electrostatic needs to be considered which reads

37 $$\begin{aligned} D_{x,x} + D_{z,z} \approx D_{z,z} = 0 \end{aligned}$$

The first term in (37) is a lower order term. One finds that the electric displacement $D_z$ is constant and it does not depend on *z*. Inserting

38 $$\begin{aligned} D_{z} = \tilde{e}_{31} \varepsilon _{xx} + \tilde{\kappa }_{33} E_{z} \end{aligned}$$

and (2) into (37) it follows

39 $$\begin{aligned} D_z \bigg |_{z=z_k}= & {} \frac{ 1}{h_k} \int _{z_{1k}}^{z_{2k}} D_{z} \, \textrm{d}z = \frac{ 1}{h_k} \int _{z_{1k}}^{z_{2k}} \left( \tilde{e}_{31} z \psi _{,x} - \tilde{\kappa }_{33} \varphi _{,z} \right) \, \textrm{d}z \nonumber \\= & {} \tilde{e}_{31} \underbrace{ \frac{ \left( z_{2k}+z_{1k} \right) }{ 2 } }_{ z_{mk} } \psi _{,x} - \frac{ \tilde{\kappa }_{33} }{h_k} \underbrace{ \left[ \varphi (x,z_{2k}) - \varphi (x,z_{1k}) \right] }_{ V^{k} } \end{aligned}$$

Reinserting Eq. (39) into Eq. (38) the electric field follows as

40 $$\begin{aligned} E_{z}^k = -\frac{ V^k(x) }{ h_k } + \frac{ \tilde{e}_{31}^k}{ \tilde{\kappa }_{33}^k } \left( z_{mk} - z \right) \psi _{,x} \end{aligned}$$

Note that the electric field consists of two parts: the dominating term directly coming from the applied voltage drop divided by the layer thickness, and secondly a lower part which depends on the rotation angle $\psi$. Similarly, the axial stress in (3) is

41 $$\begin{aligned} \sigma _{xx}^k = \left[ \tilde{C}_{11}^k + \frac{ (\tilde{e}_{31}^k)^2}{\tilde{\kappa }_{33}^k} \right] \psi _{,x} z - \frac{(\tilde{e}_{31}^k)^2}{\tilde{\kappa }_{33}^k} \psi _{,x} z_{mk} + \frac{ \tilde{e}_{31}^k}{ h_k } V^k(x) \end{aligned}$$

and the shear force and the bending moment are found be integration

42 $$\begin{aligned}{} & {} M(x) = K_M \psi _{,x} + \sum _{k} \tilde{e}_{31}^k z_{mk} b_k V^k (x) \nonumber \\{} & {} \quad \textrm{with} \quad K_M = \sum _{k} \left[ \tilde{C}_{11}^k \frac{ z_{2k}^3 - z_{1k}^3 }{3} b_k + \frac{ ( \tilde{e}_{31}^k )^2 \left( z_{2k} - z_{1k} \right) ^3 b_k }{ 12 \tilde{\kappa }_{33} } \right] \end{aligned}$$

43 $$\begin{aligned}{} & {} Q(x) = K_Q \left( \psi + w_{0,x} \right) \quad \textrm{with} \quad K_Q = \sum _{k} \tilde{C}_{55}^k \left( z_{2k} - z_{1k} \right) b_k \kappa _k \end{aligned}$$

where the bending stiffness is denoted by $K_M$, the shear stiffness by $K_Q$.

In general the last term in $K_M$, which depends on the piezoelectric coupling constant $\tilde{e}_{31}$ (see Eq. 42) can be neglected. because its contribution to the beam bending stiffness is low. For example: for a piezoelectric bimorph (PZT-5A) this piezoelectric stiffness is $3.25 \%$, for a three-layer beam with $\lambda _s = 0.8$ (using the values for steel and PZT-5A, Table 1), the piezoelectric stiffness is only $0.01 \%$.
